# H-NS Mutation-Mediated CRISPR-Cas Activation Inhibits Phage Release and Toxin Production of *Escherichia coli* Stx2 Phage Lysogen

**DOI:** 10.3389/fmicb.2017.00652

**Published:** 2017-04-18

**Authors:** Qiang Fu, Shiyu Li, Zhaofei Wang, Wenya Shan, Jingjiao Ma, Yuqiang Cheng, Hengan Wang, Yaxian Yan, Jianhe Sun

**Affiliations:** Shanghai Key Laboratory of Veterinary Biotechnology, Key Laboratory of Urban Agriculture (South), Ministry of Agriculture, School of Agriculture and Biology, Shanghai Jiao Tong UniversityShanghai, China

**Keywords:** *Escherichia coli*, H-NS, CRISPR-Cas, Stx2 phage, Shiga toxin

## Abstract

Shiga toxin-converting bacteriophages (Stx phages) carry the *stx* gene and convert nonpathogenic bacterial strains into Shiga toxin-producing bacteria. There is limited understanding of the effect that an *Escherichia coli* (*E. coli*) clustered regularly interspaced short palindromic repeats (CRISPR)-Cas adaptive immune system has on Stx phage lysogen. We investigated heat-stable nucleoid-structuring (H-NS) mutation-mediated CRISPR-Cas activation and its effect on *E. coli* Stx2 phage lysogen. The Δ*hns* mutant (MG1655Δ*hns*) of the *E. coli* K-12 strain MG1655 was obtained. The Δ*hns* mutant lysogen that was generated after Stx phage lysogenic infection had a repressed growth status and showed subdued group behavior, including biofilm formation and swarming motility, in comparison to the wild-type strain. The de-repression effect of the H-NS mutation on CRISPR-Cas activity was then verified. The results showed that *cas* gene expression was upregulated and the transformation efficiency of the wild-type CRISPR plasmids was decreased, which may indicate activation of the CRISPR-Cas system. Furthermore, the function of CRISPR-Cas on Stx2 phage lysogen was investigated by activating the CRISPR-Cas system, which contains an insertion of the protospacer regions of the Stx2 phage Min27. The phage release and toxin production of four lysogens harboring the engineered CRISPRs were investigated. Notably, in the supernatant of the Δ*hns* mutant lysogen harboring the Min27 spacer, both the progeny phage release and the toxin production were inhibited after mitomycin C induction. These observations demonstrate that the H-NS mutation-activated CRISPR-Cas system plays a role in modifying the effects of the Stx2 phage lysogen. Our findings indicated that H-NS mutation-mediated CRISPR-Cas activation in *E. coli* protects bacteria against Stx2 phage lysogeny by inhibiting the phage release and toxin production of the lysogen.

## Introduction

Shiga toxin (Stx)-producing *Escherichia coli* (STEC), especially the O157:H7 strains, are the major causes of hemorrhagic colitis and hemolytic-uremic syndrome (HUS) (Allison et al., 2003). Although some bacteriophage infections are lytic, temperate and lysogenic bacteriophages often integrate into the bacterial genome as a prophage causing a chronic infection of the host bacterium, known as lysogenic conversion (Zegans et al., 2009). It is known that Stx phage lysogenization of STEC promotes its pathogenicity and contributes to its viability in environmental conditions (Croxen et al., 2013). Stx2 production is reported to be associated with more severe diseases than strains that produce either Stx1 or a combination of Stx1 and Stx2 (Kleanthous et al., 1990; Pacheco and Sperandio, 2012; Kruger and Lucchesi, 2015). Clinically, the use of antibiotics to treat STEC infections has become controversial due to the stimulation of the lytic cycle and the concomitant toxin release through the bacterial SOS response (Croxen et al., 2013). As described previously, when phages infect bacterial cells, they must resist a range of antiviral mechanisms in order to thrive in most environments (Labrie et al., 2010). As an important bacteriophage resistance mechanism, a clustered regularly interspaced short palindromic repeats (CRISPR)-Cas system plays a vital role in preventing phage infection and multiplication.

The CRISPR-Cas system is a sophisticated adaptive immune system in prokaryotes that consists of short DNA repeats separated by 26–72 bp sequences, called spacers, which are derived from viruses and other foreign DNA (van der Oost et al., 2009; Yin et al., 2013). In addition to the CRISPR-Cas system's role in adaptive immunity, its function in regulating the behavior of bacteria has also been widely reported (Westra et al., 2014; Fu et al., 2015). Moreover, due to the high polymorphisms of CRISPR arrays, CRISPR sequences have been successfully used to classify a number of bacteria in investigations of infectious disease outbreaks (Liu et al., 2011; Louwen et al., 2014; Fu et al., 2017). Complementarity between mature CRISPR RNAs (crRNAs) and the sequences found within phages or plasmids activate Cas proteins, which target and cleave the complementary sequence, leading to the inhibition of their infection or replication (Garneau et al., 2010; Westra et al., 2012).

In strains of *Escherichia coli* (*E. coli*), such as *E. coli* K-12 MG1655, the type I-E CRISPR-Cas immune system consists of two major CRISPR loci and eight *cas* genes: *cas1, cas2, cas3*, and *casABCDE*. However, the activity of the *E. coli* CRISPR-Cas system is repressed by the heat-stable nucleoid structuring (H-NS) protein, a global transcriptional repressor. As one of the most abundant proteins in the *E. coli* nucleoid, H-NS is also widely distributed within other gram-negative bacteria (Bloch et al., 2003; Dorman, 2004, 2014). Thus, relieving H-NS-mediated repression of *cas* gene transcription may be a primary prerequisite for the CRISPR-Cas system's ability to resist phage lysogenization, lysogens, and prophage induction (Pul et al., 2010; Majsec et al., 2016). Since the Stx phage plays an important role in the dissemination of Shiga toxin genes and the emergence of new STEC strains, how the CRISPR-Cas system will perform against a Stx prophage integrated into a bacterial genome must be further elucidated. In this study, the *E. coli* O157:H7 strain Min27 was used; this pathogenic strain, which was isolated in the field, is capable of producing Shiga toxin after induction by either mitomycin C or antibiotics, such as norfloxacin and gentamicin. The activated CRISPR system's effect on the phage release, toxin production, and other biological characters of phage lysogen were determined. Since the impact of the CRISPR-Cas system on Stx2 phage lysogen is uncertain, in this study a Δ*hns* mutant of *E. coli* K-12 was generated to activate the system. Based on the general feature of spacer-protospacer matching of an *E. coli* CRISPR-Cas system, a Stx2 phage Min27 single lysogen of the Δ*hns* mutant and different lysogens that harbor the engineered CRISPRs were generated. The Stx2 phage resistant function of an H-NS-mediated *E. coli* type I-E CRISPR-Cas system was investigated by testing the lysogenization efficiency, phage release, and Shiga toxin production. The findings suggest that the CRISPR-Cas immune system impacts the biological characteristics of Stx phage lysogen in *E. coli*.

## Materials and methods

### Bacterial strains, bacteriophages, and media

The bacterial strains, bacteriophages, and plasmids used in this study are listed in Table 1. *E. coli* K-12 strain MG1655 was used in this study. The *E. coli* K-12 derivative strain, MC1061, was used as a nontoxigenic host for Stx phage infections and propagation (Allison et al., 2003). The *E. coli* strains were routinely cultured in Luria-Bertani (LB) broth (Oxoid, Basingstoke, U.K.) or LB agar (1.5%) at 37°C. The growth media were supplemented with the following antibiotics at the indicated concentrations: ampicillin (Amp), 100 μg/ml; chloramphenicol (Cm), 34 μg/ml. The Stx2 phage Min27 was obtained from *E. coli* O157:H7 strain Min27, which was isolated from the feces of a piglet with diarrhea at a swine farm in Shanghai, China (Su et al., 2010). Recombinant mutant phage Min27(Δstx::cat) was constructed using the lambda Red recombinase method with Stx2AB deleted, but carrying a chloramphenicol resistance cassette. This mutant phage was used in the latter experiment of lysogenic infection efficiency for colony-forming unit (CFU) counting of the lysogen on the Cm^R^ plate. The related primers used for mutation and verification of the recombinant phage are listed in Table 2.

**Table 1:** *****E. coli*** K-12 bacterial strains, bacteriophages, and plasmids used in this study**.

**Strains and plasmids**	**Genotype/Relevant characteristics**	**Source**
**BACTERIA**		
MG1655	Wild-type *E. coli* K-12	This study
MG1655Δ*hns*	H-NS deletion	This study
SL	MG1655ΦMin27, Wild-type Min27 phage single lysogen	This study
SLΔ*hns*	MG1655Δ*hns*ΦMin27, *hns* deletion then lysogenic infected by single wild-type Min27 phage	This study
MC1061	Indicator strain (*E. coli* K-12)	This study
FQ1	MG1655 anti-control spacer, MG1655 harboring the engineered CRISPR plasmid with control spacers; Amp^R^	This study
FQ2	MG1655 anti-Min27 spacer, MG1655 harboring the engineered CRISPR plasmid with spacers derived from the phage Min27 genome; Amp^R^	This study
FQ3	MG1655Δ*hns* anti-control spacer, MG1655 with *hns* deletion harboring the engineered CRISPR plasmid with control spacers; Amp^R^	This study
FQ4	MG1655Δ*hns* anti-Min27 spacer, MG1655 with *hns* deletion harboring the engineered CRISPR plasmid with spacers derived from the phage Min27 genome; Amp^R^	This study
FQ5	SL anti-control spacer, Wild-type Min27 phage single lysogen harboring the engineered CRISPR plasmid with control spacers; Amp^R^	This study
FQ6	SL anti-Min27 spacer, Wild-type Min27 phage single lysogen harboring the engineered CRISPR plasmid with spacers derived from the phage Min27 genome; Amp^R^	This study
FQ7	SLΔ*hns* anti-control spacer, Wild-type Min27 phage single lysogen with *hns* deletion harboring the engineered CRISPR plasmid with control spacers; Amp^R^	This study
FQ8	SLΔ*hns* anti-Min27 spacer, Wild-type Min27 phage single lysogen with *hns* deletion harboring the engineered CRISPR plasmid with spacers derived from the phage Min27 genome; Amp^R^	This study
**BACTERIOPHAGES**		
ΦMin27	Wild-type *Stx2* phage Min27; isolated from O157:H7 strain Min27	Su et al., 2010
ΦMin27(Δstx::cat)	Recombinational phage Min27 with *Stx* deletion, but carrying chloramphenicol resistance; Cm^R^	This study
**PLASMIDS**		
pKD46	Amp^R^, λRed recombinase expression	Datsenko and Wanner, 2000
pKD3	Chloramhenicol resistance cassette	Datsenko and Wanner, 2000
pCP20	Temperature sensitive replication, thermal induction of FLP recombinase synthesis; Amp^R^ and Cm^R^;	Datsenko and Wanner, 2000
pGEX-6p-1	Empty vector, Amp^R^	This study
pGEX-6p-1-CRISPR1	pGEX-6p-1 cloned with K-12 CRISPR1 loci	This study
pGEX-6p-1-CRISPR2	pGEX-6p-1 cloned with K-12 CRISPR2 loci	This study
pGEX-6p-1-anti-control spacer	Engineered CRISPR plasmid with control spacers	This study
pGEX-6p-1-anti-Min27 spacer	Engineered CRISPR plasmid with spacers derived from the phage Min27 genome	This study

*Cm^R^ and Amp^R^ represent chloramphenicol and ampicillin resistance, respectively. Strains harboring engineered CRISPR plasmid were referred to by the identifiers (FQ1–8)*.

**Table 2:** **Oligonucleotide primers used in this study**.

**Target**	**Primers (5′ → 3′, Forward/Reverse)**
***hns*** **GENE DELETION & VERIFICATION**	
*hns* Deletion	AAAAAATCCCGCCGCTGGCGGGATTTTAAGCAAGTGCAATCTACAAAAGAgtgtaggctggagctgcttc/TCTATTATTACCTCAACAAACCACCCCAATATAAGTTTGAGATTACTACAcatatgaatatcctccttag
Δ*hns* Mutant verification	GATCAGGAAATCGTCGAGGG/ATGAGCGAAGCACTTAAAAT
**RECOMBINANT MUTANT PHAGE CONSTRUCTION**	
ΦMin27(Δstx::cat) (Stx2AB)	ATGAAGTGTATATTATTTAAATGGGTACTGTGCCTGTTACTGGGTTTTTCgtgtaggctggagctgcttc/TCAGTCATTATTAAACTGCACTTCAGCAAATCCGGAGCCTGATTCACAGGcatatgaatatcctccttag
phage mutant verification (Stx)	TTCTTCGGTATCCTATTCC/TGACTCTCTTCATTCACGG
**CRISPR PLASMID CONSTRUCTION**	
pCRISPR1	CGC*GGATCC* ATCCAGTGCGCCCGGTTTA/CCG*GAATTC* ACATTAAGGTTGGTGGGTTGT
pCRISPR2	CGC*GGATCC*CTTGAGAAAGAGATAACGGG/CCG*GAATTC*TGTGACTGGCTTAAAAAATC
**Cas GENES RT-qPCR**	
*cas1*	ACCTGGCTTCCCCTTAATCC/CGCGCCATCTATTACATCGA
*cas2*	TGGCGGAAGAAGGCAATG/CTCAAATCCCGTTTCCGTATTC
*cas3*	GCAGATTTGCTTTTCCAACGA/AAATGGTGGCAACAGGAATCTC
*casA*	GCCCCGTGACGATATGGA/CGATAATTTGCCCAATGCAA
*casB*	CGCGATATCCCTGCGTTTTA/CGTGGGTTTTCCCAACCA
*casC*	GCGGCATGATGACTGAGTTG/TGATCGCATGCGCAATG
*casD*	GATACTTCTTCATTACAGGCGTTATCA/GCAACCCCGTTACAGACACA
*casE*	CTTCACCAGGGATTATGGCATT/CATGACAGCCTTCTGGTGTGTT
*tmRNA*	GGCAAGCGAATGTAAAGACTGA/CCGCGTCCGAAATTCCTA

*Underline and italic texts indicate restriction enzyme sites. GGATCC is the recognition site for BamH I and GAATTC is the recognition site for Eco R I*.

### Plasmid construction

The plasmids were constructed using standard molecular biology techniques. Standard digestion of the polymerase chain reaction (PCR) products and the vector by restriction enzymes was conducted according to the manufacturer's instructions. The CRISPR1 and CRISPR2 loci of MG1655 were amplified using specified primers, and then they were inserted into the *Bam*H I-*Eco* R I-cleaved pGEX-6p-1 vector to generate the foreign plasmids pCRISPR1 and pCRISPR2, respectively. The construction of the plasmids pCRISPR1, pCRISPR2, the p-anti-control spacer, and the p-anti-Min27 spacer, are described in the Supplementary Material.

### Δ*hns* mutant construction

The mutant with an in-frame deletion in the *hns* gene was generated using the lambda Red system, as described previously (Datsenko and Wanner, 2000; Wang et al., 2011). The *hns* gene was replaced with a chloramphenicol resistance cassette, which was amplified from plasmid pKD3 using PCR with *hns* deletion primers (Table 2). The 5′ regions of the primers (50 bp) were homologous to the corresponding flanking region of the *hns*. The PCR products were then transformed into MG1655 containing the lambda Red recombinase expression plasmid pKD46 by electroporation. After electroporation, the samples were incubated at 37°C for 1 h in Super Optimal Broth with Catabolite repression (SOC) and plated on LB agar with chloramphenicol to select the Δ*hns* mutants. The mutants were confirmed by PCR and sequenced using Δ*hns* mutant verification primers. The chloramphenicol resistance cassette was cured by transforming the pCP20 plasmid into the mutant and selecting for a chloramphenicol sensitive mutant strain, which was finally designated as MG1655Δ*hns*.

### Generation of the phage lysogens and measurements of the phage titer

To generate the Stx phage lysogens, 100 μl of filtered lytic supernatants containing phage (prepared as described above) was mixed with 100 μl of *E. coli* K-12 MG1655 cells from an overnight LB-grown culture, and the MG1655 and phage mixture was incubated overnight at 37°C in LB medium. These bacteria-phages mixtures were then struck on plates to obtain individual colonies. The individual colonies were inoculated into liquid LB medium, grown overnight, and then assayed for the presence of phage. The lytic supernatant-containing phage was serially diluted and incubated with indicator strain MC1061 (grown in LB until OD_600_ reached 0.4) plus 0.01M CaCl_2_ at 37°C for 15 min. The cells were mixed with top agar (5 ml) and poured onto LB agar plates. After incubation at 37°C for 8 h, the phage titer expressed as plaque-forming units (PFU)/ml was calculated by counting the plaques on the plate.

### Biofilm formation assay

Biofilm formation assays were performed using a previously described, 96-well plate method with some modifications (Schembri and Klemm, 2001; Skyberg et al., 2007). In brief, single lysogens were grown to stationary phase in LB at 37°C and then diluted 1:100 in LB supplemented with 5 g/L glucose. Aliquots of 200 μl for each dilution were dispensed per well into a microtiter plate. The negative-control wells contained uninoculated medium. The plates were cultured aerobically at 37°C without shaking for 24 h. The medium of the plates was then poured off, and the plates were triple-washed with sterile phosphate-buffered saline (PBS). The microplates were then stained with 200 μl of 1% (w/v) crystal violet for 30 min, washed three times with PBS to remove the unbound crystal violet dye, and air dried for 1 h. After drying, the adherent cells were re-solubilized with 200 μl of 30% acetic acid. The absorbance was measured at 550 nm in a microplate (ELISA) reader (BioTek, ELx800). All tests were carried out three times independently, and the results were analyzed statistically.

### Swarming mobility assay

Swarming assays were performed as reported previously (Toutain et al., 2005; Su et al., 2010). The swarming motility of the single lysogens was tested by inoculating 5 μl of an overnight, LB-grown culture onto a swarming plate followed by incubation at 37°C for 48 h. A semisolid medium containing 0.5% agar was supplemented with 0.5% D-(+)-glucose. The swarming motility plates were prepared and used at the same day, after solidification of agar, they were dried for 5 min under sterile air and then inoculated.

### Quantitative real-time reverse transcription-PCR

The bacteria were grown to the logarithmic phase, and RNA was isolated using the E.Z.N.A. bacterial RNA isolation kit (Omega, Beijing, China) according to the manufacturer's instructions. Contaminated DNA was removed from the samples with RNase-free DNase I (Thermo Scientific, Waltham, MA, USA), and cDNA synthesis was performed using the Reverse Transcription System (Promega, Madison, WI, USA) according to the manufacturer's instructions. Quantitative real-time reverse transcription-PCR (RT-qPCR) was performed to determine the transcription levels of the *cas* genes using SYBR Green MasterMix (DBI Bioscience, Ludwigshafen, Germany) and gene-specific primers (Table 2), and the data were normalized with the housekeeping gene tmRNA transcript. The relative fold change was calculated using the threshold cycle (2^−ΔΔCT^) method.

### Transformation efficiency assay

The *E. coli* MG1655 and MG1655Δ*hns* cultures were incubated at 37°C until an OD_600_ of 0.5–0.6 was reached. The bacteria were then centrifuged at 4,000 × g at 4°C, the supernatants were disposed, and the bacteria were resuspended in 1 ml ice-cold 10% glycerol, and then transferred to a 1.5 ml tube. After additional washing steps, the cells were suspended in 200 μl ice-cold 10% glycerol. The bacterial cells (50 μL) were then mixed in an ice-cold 0.2 mm electroporation cuvette (Bio-Rad) with 2 μg of the pVEC, pCRISPR1, or pCRISPR2 plasmids. The mixture was pulsed in a Bio-Rad MicroPulser at 200 Ω, 25 μF, and 1.8 kV. Immediately after the pulse, 1 ml of SOC broth was added, and the cells were aerated for 2 h at 37°C. Various dilutions of the reaction were plated on LB agar plates supplemented with 100 μg/ml Ampcillin. The plates were incubated overnight at 37°C. The colonies emerging on the selection plates were counted, and the CFU number per microgram plasmid was calculated, accordingly.

### Lytic and lysogenic infection efficiency assay

The *E. coli* MG1655 and MG1655Δ*hns* cultures harboring the engineered CRISPRs (containing either the control spacer or the Min27 spacer) (FQ1, FQ2, FQ3, and FQ4 in Table 1) were incubated at 37°C until an OD_600_ of 0.5–0.6 was reached. All the strains were normalized to OD_600_ = 0.6, and the CFU was also counted and calculated on the Amp^R^ plate. For lytic infection, aliquot supernatants containing infectious Min27 phage particle was added to 5 ml of the normalized cultures and then cultured aerobically at 37°C with shaking for 12 h, respectively. The cultures were then centrifuged at 4,000 × g for 5 min, and the supernatants were used to determine the phage titer via PFU assay. The efficiency of the plating (EOP) (EOP = phage titer on test bacterium/phage titer on indicator bacterium MC1061) was also measured. For the lysogenic infection, the recombinant mutant phage Min27(Δstx::cat), which carries a chloramphenicol resistance cassette, was used. A total of 100 μl of the filtered phage Min27(Δstx::cat) was mixed with 100 μl of the *E. coli* MG1655 and MG1655Δ*hns* cultures harboring the engineered CRISPRs, and the mixtures were incubated overnight at 37°C. These bacterium-phage mixtures were then struck on Amp^R^ (lysogens and non-lysogens cells) or Amp^R^ plus Cm^R^ (lysogens) plates to obtain individual colonies and to calculate the CFUs.

### Phage release of the lysogens

The lysogens harboring the engineered CRISPR (FQ5, FQ6, FQ7, and FQ8) and the control strains were induced to release the phage by exposing the mid-exponential-phase cultures (all normalized to OD_600_ = 0.6) of the representative ampicillin-resistant colonies to mitomycin C (0.5 μg/ml) (Sigma) (4) for 12 h. The progeny phage titer was determined using the PFU assay described above. Furthermore, the lysogen survival rate after mitomycin C induction was also calculated using CFU counting.

### Vero cell viability assay

The Vero cells were plated at 10^4^/well on 96-well plates in Dulbecco's Modified Eagle Medium (DMEM) containing 10% fetal bovine serum, and then incubated overnight at 37°C under 5% CO_2_. Four lysogens FQ5, FQ6, FQ7, and FQ8 were induced to release the phage and Shiga toxin by exposing the mid-exponential-phase cultures (all normalized to OD_600_ = 0.6) of the representative ampicillin-resistant colonies to mitomycin C (0.5 μg/ml) (Sigma) (4) for 12 h. Serial 10-fold dilutions of the filtered (0.22 μm filter) supernatants in DMEM medium were prepared. Dilutions (10^−1^–10^−4^) were added to the cell monolayer (100 μl/well) and incubated for 48 h at 37°C under 5% CO_2_. The viability of the Vero cells was determined via crystal violet staining. All data was represented as the average of triplicate assays. A lysogen without cytotoxic effect, MG1655ΦMin27(Δstx::cat), was used as a negative control.

### Western blot analysis

A total of 10 ml the lysogens harboring the engineered CRISPR (FQ5, FQ6, FQ7, and FQ8) were induced to release the phage and Shiga toxin by exposing the cultures (all normalized to OD_600_ = 0.6) of the representative ampicillin-resistant colonies to mitomycin C (0.5 μg/ml) for 12 h. The filtered supernatants were collected and then subjected to saturated ammonium sulfate precipitation. The mixture of the supernatants and the saturated ammonium sulfate (1:1) was gently shaken at 4°C for 6 h. The resulting precipitation was dissolved in 1 ml PBS after centrifugation at 10,000 × g for 10 min. The prepared samples were subjected to sodium dodecyl sulfate (SDS)-polyacrylamide gel electrophoresis (PAGE), and then examined via Western blot analysis using the Stx2 monoclonal antibody (MAb) 11E10 (Santa Cruz, Dallas, USA), as previously described (Smith et al., 2009).

### Statistical analyses

The reported differences were analyzed using either Student's *t*-test (**Figure 2B**) or one-way analysis of variance (ANOVA) (**Figure 6A**). The analyses were conducted using GraphPad Prism 5 software. A *P* < 0.05 was considered to be statistically significant.

## Results

### H-NS deletion mediates *E. coli* K-12 CRISPR-Cas activation

**Figure 1:**
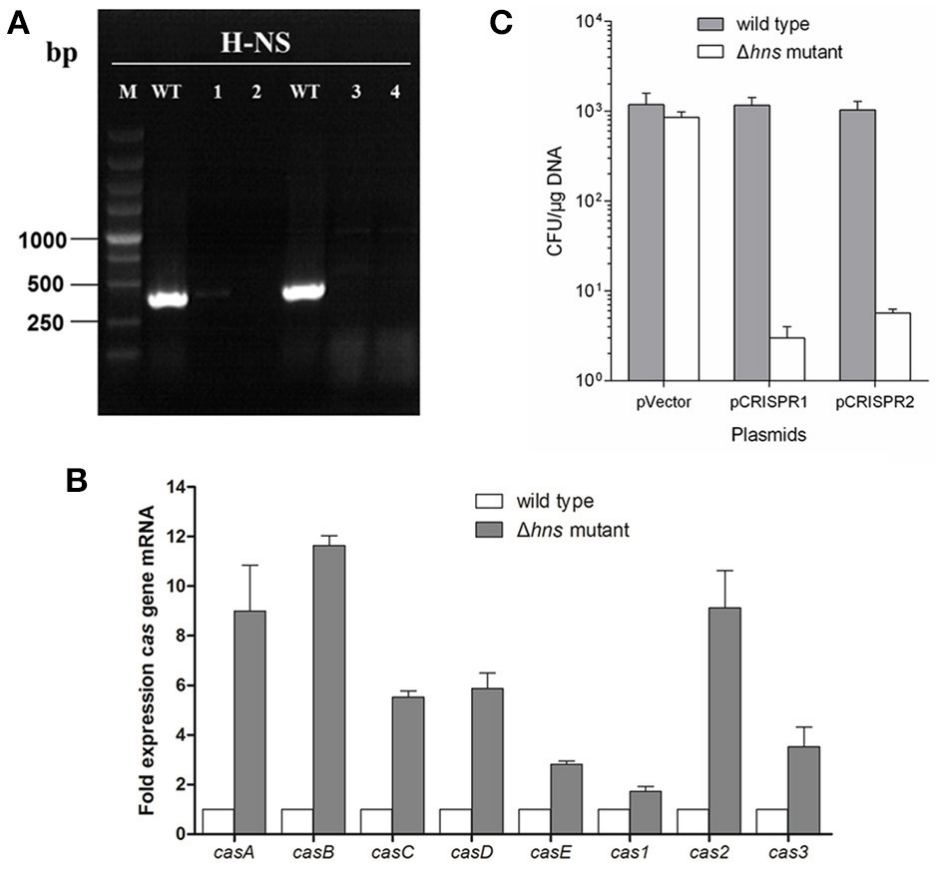
**H-NS deletion mediates ***E. coli*** K-12 CRISPR-Cas activation. (A)** The mutant with an in-frame deletion in the *hns* gene was generated and verified by PCR. Line M indicates a DL5000 DNA marker; Line WT indicates the wild-type strain. Lines 1, 2, 3, and 4 indicate four independent mutant colonies. The results show that the *hns* genes of the four mutants were successfully deleted. Line 2 was chosen as the Δ*hns* mutant for future experiments. **(B)** *Cas* gene transcription levels of the wild-type (MG1655) and Δ*hns* mutant (MG1655Δ*hns*) strains in the mid-exponential growth phase were examined using the RT-qPCR method. **(C)** Transformation efficiency of the foreign plasmids encoding the homologous spacer with the *E. coli* CRISPR loci was determined. Plasmids harboring two CRISPR loci, CRISPR1 and CRISPR2, as well as an empty vector, were transformed into the Δ*hns* and wild-type strains, and the transformation efficiencies were calculated, respectively.

It has been reported that, under laboratory conditions, transcription of the *Cascade* genes in the *E. coli* CRISPR-Cas system is repressed by H-NS (Edgar and Qimron, 2010; Pul et al., 2010; Majsec et al., 2016). In order to investigate the interactions between CRISPR-Cas and phage lysogen, a mutant with *hns* gene in-frame deletion was generated (Figure 1A). To study the effect of *hns* deletion on CRISPR-Cas activation and *cas* gene expression, the transcription levels of the wild-type MG1655 and the Δ*hns* strain *cas* genes in the mid-exponential growth phase were examined via real-time quantitative polymerase chain reaction (RT-qPCR). The Δ*hns* strain revealed a significant upregulation of the transcriptions of *casABCDE, cas1, cas2*, and *cas3* (average 5-fold difference) than the wild-type strain (Figure 1B). These results are consistent with the findings reported in previous studies (Westra et al., 2010), indicating that *cas* gene transcription is closely associated with H-NS.

It has been demonstrated that the CRISPR-Cas system provided protection against invading mobile genetic elements (MGEs), such as plasmids and phages (Barrangou and Marraffini, 2014; Barrangou, 2015). To further verify that H-NS deletion mediates CRISPR-Cas activation, the transformation efficiency of the foreign plasmids that carry the homologous spacer with *E. coli* CRISPR loci was determined. Plasmids harboring two CRISPR loci, known as CRISPR1 and CRISPR2, as well as an empty vector, were transformed into the Δ*hns* mutant and wild-type strains, and the transformation efficiencies were calculated, respectively. The plasmid containing the CRISPR1 sequences (with the same protospacer against the CRISPR1 loci) transformed into the Δ*hns* mutant with an efficiency that was approximately 0.35% of the efficiency of the empty vector, but no obvious difference in transformation efficiency was observed in the wild-type strain (Figure 1C). Similarly, transformation of the plasmids containing the CRISPR2 protospacer into MG1655Δ*hns* was reduced by around 90% in comparison to the empty vector. In contrast, no reduction in the transformation efficiency was observed for the plasmids when they were introduced into the wild-type strain (Figure 1C). These results clearly demonstrate that the proposed *E. coli* CRISPR-Cas system is active against foreign plasmids after relieving the *hns* repression.

### H-NS regulates the group behavior of the *E. coli* Stx2 phage lysogen

**Figure 2:**
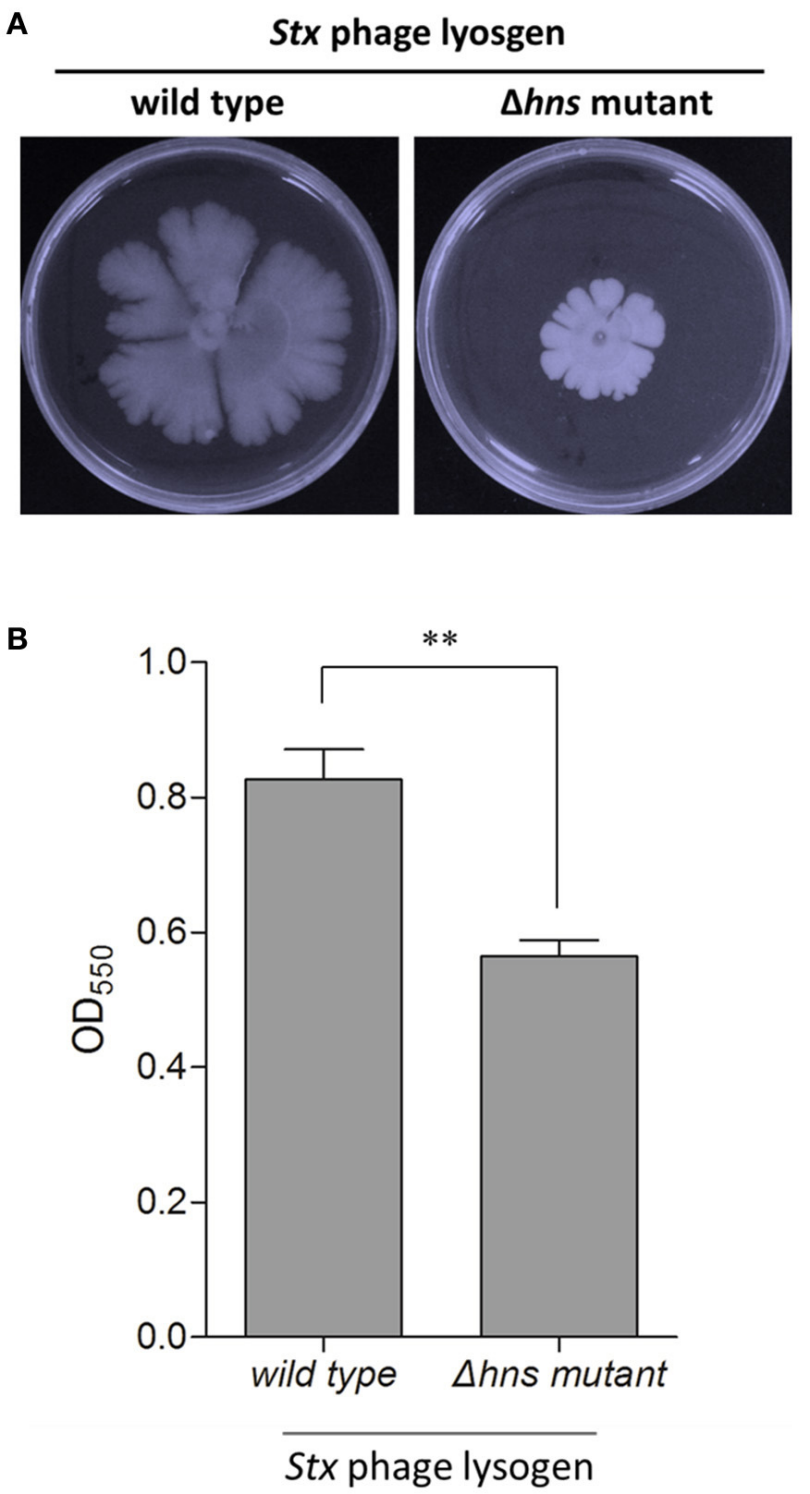
**H-NS regulates the group behavior of the ***E. coli*** Stx2 phage lysogen**. The biofilm formation and swarming motility ability of the Stx2 phage lysogenic wild-type and Δ*hns* mutant strains were assessed. **(A)** Swarming motility of the single lysogens was tested by inoculating 5 μl of an overnight, LB-grown culture onto a swarming plate followed by incubation at 37°C for 48 h. **(B)** Biofilm formation was assessed by crystal violent staining of the biofilms grown in microplate wells for 24 h. The data is representative of at least three independent experiments. Student's *t*-tests were used to determine statistically significant differences (*p* < 0.05). ^*^p < 0.05; ^**^p < 0.01.

To access the possible role of H-NS mutation-mediated CRISPR-Cas immunity in regulating these bacterial group behaviors, we evaluated the biofilm formation and swarming motility ability of the Stx2 phage lysogenic wild-type and Δ*hns* mutant strains. The results showed that the swarming mobility of the Δ*hns* mutant lysogen was extremely inhibited in comparison to the wild-type Min27 phage lysogen (Figure 2A). In bacteria lysogenized with bacteriophage Min27, the Δ*hns* mutant lysogen showed decreased biofilm formation ability in comparison to the wild-type Min27 phage lysogen (Figure 2B). Biofilm formation is another type of bacterial group behavior. The results suggest that lysogeny by Stx phage Min27 interferes with at least two *E. coli* group behaviors. It was also observed that lysogenic infection of *Pseudomonas aeruginosa* PA14 with bacteriophage DMS3 inhibits bacterial group behaviors, such as biofilm formation and swarming motility, which were later demonstrated to be associated with the CRISPPR-Cas system function (Zegans et al., 2009). These observations indicate that the activated *E. coli* CRISPR-Cas system may also be involved in inhibiting the group behavior of the Stx2 phage lysogen.

### Activated CRISPR-Cas inhibits phage replication

**Figure 3:**
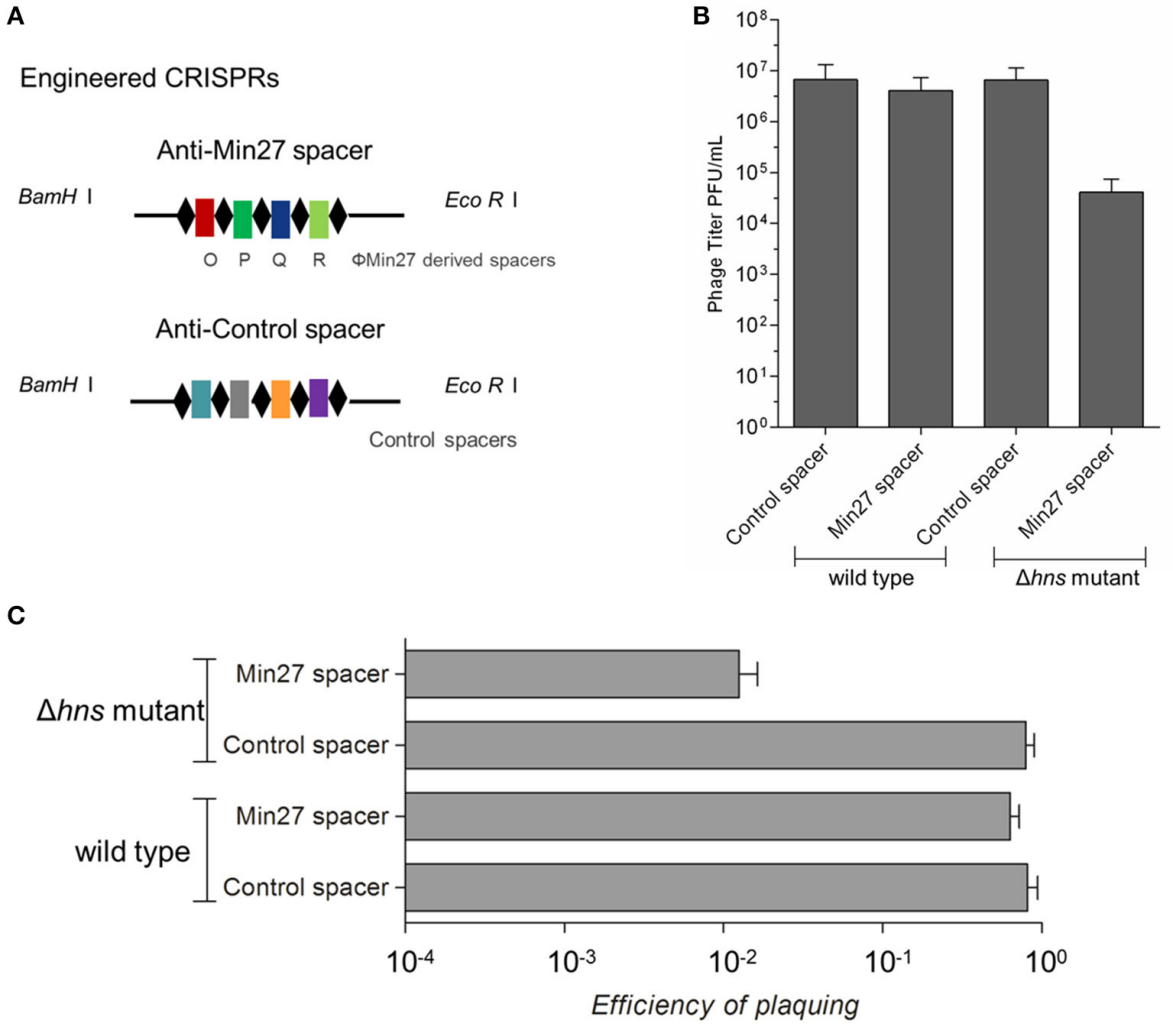
**Activated CRISPR-Cas inhibits phage replication. (A)** Schematic diagram of the engineered CRISPRs. The sequence of the anti-Min27 spacers is taken from the genome of the Stx phage Min27. Repeats are the same as the CRISPR loci in MG1655. The spacers are homologous protospacers (32 bp) in the phage Min27 genes *O, P, Q*, and *R*, respectively. **(B)** Four strains, FQ1, FQ2, FQ3, and FQ4, were used to assess their ability to hamper phage propagation. **(C)** The effect of the CRISPR-Cas system on phage propagation in the four strains was measure by efficiency of plating (EOP) assay. (EOP = phage titer on test bacterium/phage titer on indicator bacterium MC1061). All assays were conducted at least three times.

To evaluate the possible function of CRISPR-Cas-associated immunity in inhibiting the replication of phages in the *E. coli* K-12 stains, four strains of the wild-type and Δ*hns* mutant harboring the engineered CRISPRs (containing either the control spacer or the Min27 phage-derived spacer) were generated: FQ1, FQ2, FQ3, and FQ4. These were used to detect their ability to hamper phage propagation (Figure 3A). After infecting the strains with the Stx2 phage Min27, progeny phage titer in the *hns-*deleted strain harboring the Min27 phage-derived spacer showed a nearly 100-fold decrease in comparison to the other strains, 12 h post-infection (Figure 3B). Further evidence showing that H-NS mediates CRISPR-based immunity against the Stx2 phage was obtained using the efficiency of plaquing (EOP) assay. After introducing the Min27-derived spacer CRISPR plasmid into the Δ*hns* mutant (FQ4), it reduced the sensitivity to the Stx2 phage Min27 by 100-fold, in contrast to the wild-type strain or the strains harboring the control spacer CRISPR plasmid (FQ1, FQ2 and FQ3) (Figure 3C). These data show that the *hns* mutation containing the Min27 phage-derived spacer plasmid led to a dramatic CRISPR-Cas-mediated inhibition of phage Min27 replication (Figure 3A), and it also contributed to a reduction in the efficiency of plaquing (Figure 3C).

### Activated CRISPR-Cas represses phage lysogenic infection

**Figure 4:**
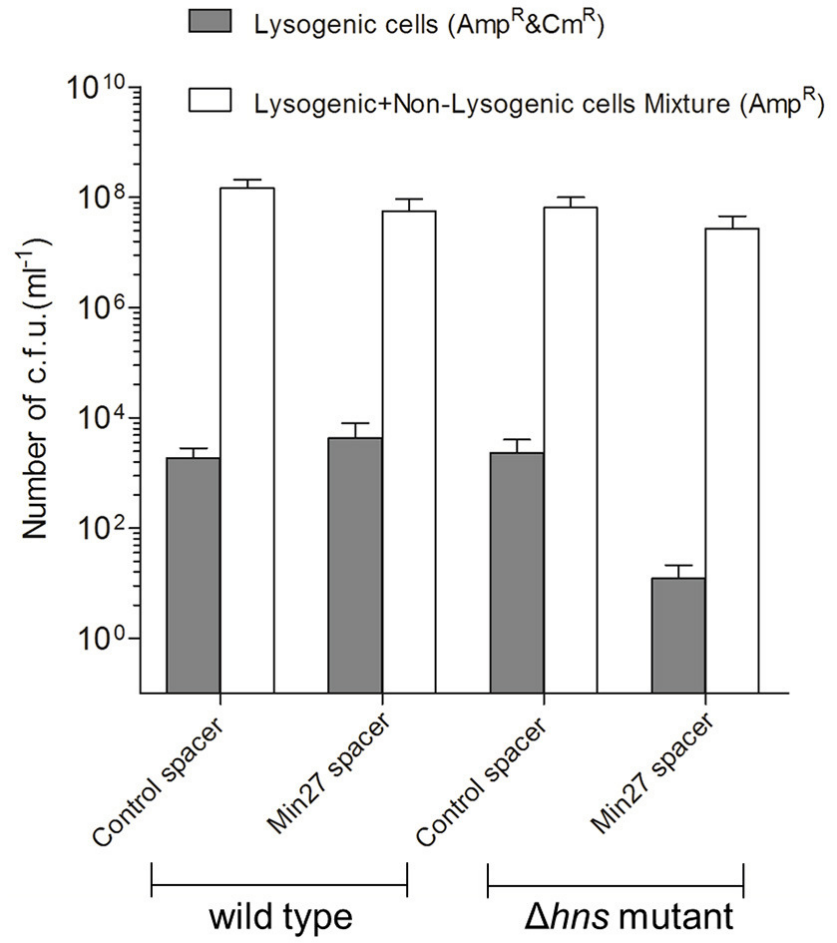
**Activated CRISPR-Cas represses the Stx phage lysogenic infection**. The recombinant mutant phage Min27(Δstx::cat) was used to lysogenically infect wild-type and Δ*hns* mutant MG1655 strains harboring the engineered CRISPRs (containing either the control spacer or the Min27 phage-derived spacer) plasmids (FQ1, FQ2, FQ3, and FQ4). A total of 100 μl of filtered phage Min27(Δstx::cat) was mixed with 100 μl of the cultures of the four indicated strains, and the mixtures were incubated overnight at 37°C. These bacterium-phage mixtures were then plated onto Amp^R^ (lysogenic and non-lysogenic cells) or Amp^R^ plus Cm^R^ (lysogens) plates to obtain individual colonies and to calculate the CFU count.

To further elucidate the immune mechanism of CRISPR-Cas against the Stx2 phage, which is mediated by H-NS, the recombinant mutant phage Min27(Δstx::cat) was used to lysogenically infect the wild-type and Δ*hns* mutant, each of which were harboring the engineered CRISPRs (FQ1, FQ2, FQ3, and FQ4), respectively. Then, the lysogenic infection efficiency of the Stx2 phage against the above strains was evaluated by counting the CFUs. The stx2 gene from the toxin operon was interrupted by a chloramphenicol resistance cassette (see Materials and Methods), which is convenient for counting the CFUs. After infection, the bacterium-phage mixtures were serial-diluted and then plated onto Amp^R^ (lysogenic and non-lysogenic cells) or Amp^R^ plus Cm^R^ (lysogenic cells) plates to obtain individual colonies and to calculate the CFU count, respectively. As shown in Figure 4, there was no significant difference among the CFU counts for the lysogenic and non-lysogenic cells grown on the Amp^R^ plates, which may indicate that the phage had initiated the lysogenic cycle, so that it did not result in a great mass of bacterial lysis. However, for the Amp^R^ plus Cm^R^ plates, on which the lysogenic cells were screened and counted, the efficiency of the lysogenic infection against FQ4 was greatly reduced (~10^2^ times) in comparison to the other three strains (FQ1, FQ2, and FQ3) (Figure 4). These data imply that the activated CRISPR-Cas system in the Δ*hns E. coli* MG1655 strain strongly dampens the lysogenic infection of the Stx2 phage.

### Phage release of the lysogen was targeted by CRISPR-Cas system

**Figure 5:**
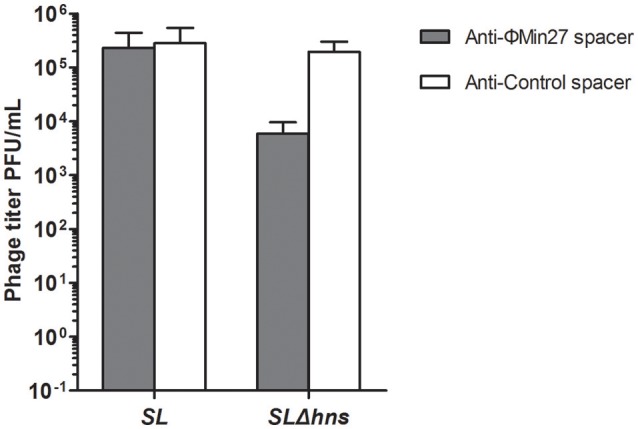
**Phage release of the lysogens was determined by PFU assay**. Mitomycin C was added to the culture of lysogens to induce phage release. SL indicates the Stx phage Min27 single lysogen; SLΔ*hns*, Stx phage Min27 single lysogen with *hns* deletion.

To evaluate the effect of the CRISPR-Cas system on the phage release of the lysogens, mitomycin C was added to the culture to induce phage release. Then, the titers of the supernatants containing the phages were determined. As shown in Figure 5, the phage titer produced by the Min27 phage lysogen with the CRISPR plasmid carrying the anti-Min27 spacers (FQ8) was approximately 100-times lower than the Δ*hns* lysogen with the control spacer (FQ7). It was also observed that the phage titer of the wild-type lysogen harboring either Min27 spacer (FQ6) or the control spacer (FQ5) was not remarkably affected. This result indicates that the phage release of the lyosgen harboring the Min27 spacers is targeted by the H-NS-mediated CRISPR activity. Thus, the presence of spacers derived from the phage Min27 is harmful for Δ*hns* mutant lysogen in the phage release after mitomycin C induction.

### Shiga toxin expression of the lysogen was blocked by the CRISPR-Cas system

**Figure 6:**
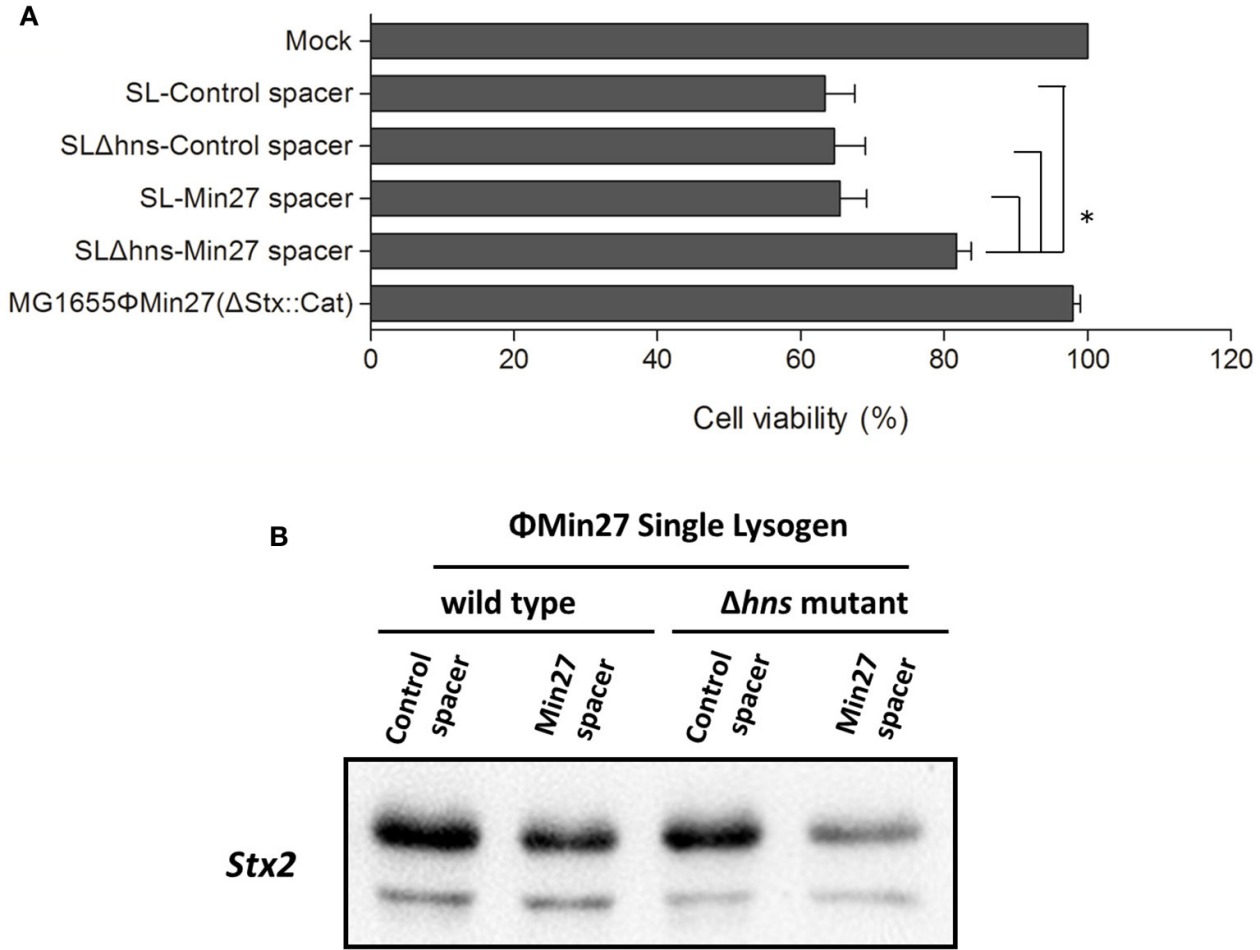
**Shiga toxin production of the lysogen was measured using a Vero cell viability assay and Western blot analysis. (A)** Serial 10-fold dilutions of the filtered supernatants were added to the cell monolayer (100 μl/well) and incubated for 48 h at 37°C under 5% CO_2_. The viability of the Vero cells was determined by crystal violet staining. Lysogen, MG1655ΦMin27(Δstx::cat), which has an inconspicuous cytotoxic effect on Vero cells, was used as a negative control. One-way analysis of variance (ANOVA) was used for data analysis (*p* < 0.05 is considered to be statistically significant) (^*^p < 0.05). **(B)** The filtered supernatants were collected and then subjected to saturated ammonium sulfate precipitation. The prepared samples were subjected to SDS-polyacrylamide gel electrophoresis (PAGE), and then examined by Western blot analysis using Stx2 monoclonal antibody (MAb) 11E10.

Since the activated CRISPR-Cas system exerts an influence on the phage release of the lysogen, the Shiga toxin expression of the lysogen after induction might also be affected by the CRISPR activity. To verify the effect of CRISPR-Cas, the cytotoxic effect of the supernatants on Vero cells was measured and the Shiga toxin expression was evaluated via Western blot analysis using Stx2 monoclonal antibody (11E10), respectively. To evaluate the potential damage induced by Shiga toxin, the cytotoxicity was evaluated in Vero cells. The Vero cell viability assay results showed that, although the cell death in the Δ*hns* mutant lysogen containing the Min27 spacer (FQ8) was only about 20%, on average, the cell death caused by the culture supernatants from the other three lysogens (FQ5, FQ6, and FQ7) was approximately 40%. In comparison to the other lysogens, the cytotoxic effect of FQ8 was significantly alleviated (Figure 6A). As a control, the supernatants of the recombinant mutant Min27 phage lysogen, MG1655ΦMin27(Δstx::cat) with no Shiga toxin expression, showed inconspicuous toxicity to the cells. This confirms that the cell death was mainly caused by the Shiga toxin. Likewise, Western blot analysis of the Shiga toxin expression also demonstrated that, with *hns* deletion, the reduction of Shiga toxin production was greater in the single lysogen harboring the Min27 spacer than it was in the other lysogens, indicating that H-NS mutation-mediated CRISPR-Cas activity was involved in blocking Shiga toxin expression (Figure 6B).

## Discussion

H-NS is an abundant bacterial protein with DNA and RNA binding activity, and it has been found to be involved in the process of gene transcription silencing (Brescia et al., 2004; Dorman, 2004, 2014). Moreover, the effect of the bacterial H-NS protein on gene expression is overwhelmingly negative (Dorman, 2004). In *E. coli*, H-NS inhibits the transcription of the precursor crRNA by binding to the AT-rich CRISPR leader promoter to repress the immune activity of type I-E CRISPR-Cas against phages and plasmids (Pul et al., 2010; Medina-Aparicio et al., 2011). Pul et al. (2010) reported that *casA* and CRISPR expression is repressed by H-NS in *E. coli*. In this study, we investigated the relationship between the Stx2 phage lysogen and *E. coli* CRISPR-Cas immune activity based on an H-NS-deficient strain. Since we did not obtain an *E. coli* K-12 Δ*hns* mutant strain from the Keio collection, an *hns* gene in-frame deletion mutant was generated. The transcription levels of the *cas* gene were upregulated in the H-NS deficient strain in comparison to the wild-type strain (Figure 1B), which is similar to the findings reported in a previous study (Pul et al., 2010). This result indicates that, by relieving the H-NS repression, the *E. coli* CRISPR-Cas system seems to be activated. Moreover, the results of the transformation efficiency of the foreign plasmids carrying the homologous spacer into both the wild-type and the Δ*hns* mutant strains further confirmed that H-NS deletion mediates CRISPR-Cas activation (Figure 1C). These data provide experimental evidence that the Δ*hns* mutant strain, which we generated, was CRISPR-activated, and it can be used in further CRISPR-Cas associated investigations.

In addition to the function of adaptive immunity, the CRISPR-Cas system has also been reported to be involved in modulating other processes, such as the genetic regulation of group behavior and virulence, DNA repairing, and genome evolution (Westra et al., 2014). It is important to note that Zegans et al. (2009) observed that lysogenic infection of *Pseudomonas aeruginosa* PA14 with bacteriophage DMS3 inhibits biofilm formation and swarming motility, both of which are important bacterial group behaviors. This suggests that CRISPR-Cas plays a role in modifying the effects of lysogeny on *Pseudomonas aeruginosa* (Zegans et al., 2009). Interestingly, the biofilm formation and swarming motility ability of the Stx2 phage lysogenic Δ*hns* mutant strains were significantly decreased in comparison to the wild-type lysogen. Although additional investigation is necessary, it is possible that the type I-E CRISPR-Cas system may exert some influence on the regulation of group behavior in Stx2 phage lysogens.

In *E. coli*, and many other types of bacteria, a specialized ribonucleoprotein complex, known as the Cascade, confers immunity by an interference reaction between the CRISPR-transcript RNA (crRNA) and the invader DNA sequences, known as protospacers. The Cascade recognizes the invader DNA via short protospacer adjacent motif (PAM) sequences and crRNA-DNA complementarity (Majsec et al., 2016). Given that the CRISPR system acts against the prophage and the CRISPR system was activated when the *hns* was deleted, it could protect lysogens from prophage induction and Shiga toxin production. In this study, according to the spacer matching requirements of the CRISPR-Cas interference process, based on the PAM sequence, four protospacers were selected from the phage Min27 genome to construct an engineered CRISPR plasmid. The results show that, in the single lysogen of Min27 phage, which was harboring the phage genome-derived spacers, both progeny phage release and Shiga toxin production were inhibited with the *hns* deletion in comparison to the control lysogen. It has also been shown that the lysogenic infection efficiency of recombinant mutant phage Min27(Δstx::cat) against FQ4 (Δ*hns* mutant harboring Min27 phage spacers) is much lower than it is in FQ1, FQ2, and FQ3 (other three stains). A previous study reported that even low CRISPR activity is still toxic to lysogenic cells carrying targeted spacers (Edgar and Qimron, 2010). Therefore the reduction of phage release and Shiga toxin production might result from CRISPR-activity-meditated bacterial cell death. The bacterial death may be a result of the targeting and neutralization of prophage DNA, leading to bacterial DNA destruction by CRISPR-Cas activity (Edgar and Qimron, 2010). In fact, the survival rate of the lysogens harboring the engineered CRISPR plasmids after mitomycin C induction was detected in our study. It was shown that the survival rate of FQ8 was much lower than the survival rate of FQ7 (data not shown). Although it is possible that the CRISPR system may affect the level of the *CI* repressor in the cell, which leads to the induction of the prophage, this possibility was ruled out experimentally (Edgar and Qimron, 2010). Another possible explanation is that, in the case of Stx phage, the induction of Stx prophages has been shown to be controlled by the *RecA*-a regulator of the SOS bacterial response (Kruger and Lucchesi, 2015; Sheng et al., 2016). It has been shown that the interaction of the Type I-F CRISPR system with the phage protospacer induces expression of the SOS-regulated phage-related genes through the activity of the nuclease Cas3 and subsequent *RecA* activation (Heussler et al., 2015). In this present study, the H-NS-mediated CRISPR-Cas activity might also affect the *RecA* gene expression in order to regulate Stx prophage induction and Shiga toxin production. A previous study indicated that since bacteria can use quorum sensing to regulate their antiphage activities, they could specifically upregulate their defense mechanisms to avoid infection (Høyland-Kroghsbo et al., 2013). Another possibility is that quorum sensing played an essential role in regulating the type I (type I-E/I-F) CRISPR-Cas system, which may affect phage lysogen biological characteristics (Patterson et al., 2016; Høyland-Kroghsbo et al., 2017). Further study is needed to determine the mechanism by which intermediates regulate the CRISPR-Cas system on Shiga toxin production, and to verify if quorum sensing participates in controlling CRISPR adaptive immunity to Stx phage lysogen.

## Author contributions

JS, YY, and QF conceived and designed the experiments; QF and SL performed the experiments; QF, ZW, JM, and YC analyzed the data; QF, YY, WS, and HW contributed reagents and materials; QF and JS wrote the manuscript.

### Conflict of interest statement

The authors declare that the research was conducted in the absence of any commercial or financial relationships that could be construed as a potential conflict of interest.
