# Supplementary material for: H-NS Mutation-Mediated CRISPR-Cas Activation Inhibits Phage Release and Toxin Production of Escherichia coli Stx2 Phage Lysogen
Source: Front Microbiol. 2017 Apr 18;8:652. doi: 10.3389/fmicb.2017.00652 (PMC5394155; doi:10.3389/fmicb.2017.00652)
Supplement: Supplementary file 1 [file DataSheet1.DOC]

Supplemental material

1. The CRISPR1 and CRISPR2 loci of MG1655 were amplified using specified primers, and then they were inserted into the *Bam*H I-*Eco* R I-cleaved pGEX-6p-1 vector to generate the foreign plasmids pCRISPR1 and pCRISPR2, respectively.

CRISPR1 Sequence

CGC*GGATCC*ATCCAGTGCGCC***CGGTTTATCCCCGCTGATGCGGGGAACAC***CAGCGTCAGGCGTGAAATCTCACCGTCGTTGC***CGGTTTATCCCTGCTGGCGCGGGGAACTC***TCGGTTCAGGCGTTGCAAACCTGGCTACCGGG***CGGTTTATCCCCGCTAACGCGGGGAACTC***GTAGTCCATCATTCCACCTATGTCTGAACTCC***CGGTTTATCCCCGCTGGCGCGGGGAACTC***CCGGGGGATAATGTTTACGGTCATGCGCCCCC***CGGTTTATCCCCGCTGGCGCGGGGAACTC***TGGGCGGCTTGCCTTGCAGCCAGCTCCAGCAG***CGGTTTATCCCCGCTGGCGCGGGGAACTC***AAGCTGGCTGGCAATCTCTTTCGGGGTGAGTC***CGGTTTATCCCCGCTGGCGCGGGGAACTC***TAGTTTCCGTATCTCCGGATTTATAAAGCTGA***CGGTTTATCCCCGCTGGCGCGGGGAACTC***GCAGGCGGCGACGCGCAGGGTATGCGCGATTCG***CGGTTTATCCCCGCTGGCGCGGGGAACTC***GCGACCGCTCAGAAATTCCAGACCCGATCCAAA***CGGTTTATCCCCGCTGGCGCGGGGAACTC***TCAACATTATCAATTACAACCGACAGGGAGCC***CGGTTTATCCCCGCTGGCGCGGGGAACTC***AGCGTGTTCGGCATCACCTTTGGCTTCGGCTG***CGGTTTATCCCCGCTGGCGCGGGGAACTC***TGCGTGAGCGTATCGCCGCGCGTCTGCGAAAG***CGGTTTATCCCCGCTGGCGCGGGGAACTC***TCTAAAAGTATACATTTGTTCTTAAAGCATTTTTTCCCATAAAAACAACCCACCAACCTTAATGT*GAATTC*CGG

CRISPR2 Sequence

CGC*GGATCC*CTTGAGAAAGAGATAACGGGTTATATGGT***GGTTTATCCCCGCTGGCGCGGGGAACTC***GACAGAACGGCCTCAGTAGTCTCGTCAGGCTCC***GGTTTATCCCCGCTGGCGCGGGGAACAC***CTGTTTTCGCAAATCTATGGACTATTGCTATTC***GGTTTATCCCCGCTGGCGCGGGGAACAC***GGGCGCACGGAATACAAAGCCGTGTATCTGCTC***GGTTTATCCCCGCTGGCGCGGGGAACAC***TGGCTCTGCAACAGCAGCACCCATGACCACGTC***GGTTTATCCCCGCTGGCGCGGGGAACAC***GAAATGCTGGTGAGCGTTAATGCCGCAAACACA***GGTTTATCCCCGCTGGCGCGGGGAACAC***ATTACGCCTTTTTGCGATTGCCCGGTTTTTGCC***GGTTTATCCCCGCTGGCGCGGGGAACAC***TCTAAACATAACCTATTATTAATTAATGATTTTTTAAGCCAGTCACA*GAATTC*CGG

Note: Repeats are bolded and italicized, spacers are colored.

2. Engineered CRISPRs Construction

Anti-Control spacers

The sequence of the control spacers is taken from (Brouns et al., 2008).

CGC*GGATCC*GGCGCGCCATGGAAACAAAGAATTAGCTGATCTTTAATAATAAGGAAATGTTACATTAAGGTTGGTGGGTTGTTTTTATGGGAAAAAATGCTTTAAGAACAAATGTATACTTTTAGA***CGGTTTATCCCTGCTGGCGCGGGGAACTC***CTTTCGCAGACGCGCGGCGATACGCTCACGCA***CGGTTTATCCCTGCTGGCGCGGGGAACTC***CAGCCGAAGCCAAAGAATTCGCCGAACACGCT***CGGTTTATCCCTGCTGGCGCGGGGAACTC***GGCTCCCTGTCGGTTGTAATTGATAATGTTGA***CGGTTTATCCCTGCTGGCGCGGGGAACTC***TTTGGATCGGGTCTGGATCCTCTGAGCGGTCG***CGGTTTATCCCTGCTGGCGCGGGGAACTC***CAGCTCCCATTTTCAAACCCATCAAGACGCGGTACCTTAATTAA*GAATTC*CGG

Anti-Min27 spacers

The sequence of the anti-Min27 spacers is taken from the genome of the Stx phage Min27.  Repeats are the same as the CRISPR loci in MG1655. The spacers have homologous protospacers (32bp) in the phage Min27 genes *O*, *P*, *Q*, and *R*, respectively.

CGC*GGATCC*GGCGCGCCATGGAAACAAAGAATTAGCTGATCTTTAATAATAAGGAAATGTTACATTAAGGTTGGTGGGTTGTTTTTATGGGAAAAAATGCTTTAAGAACAAATGTATACTTTTAGA***CGGTTTATCCCTGCTGGCGCGGGGAACTC***GGCGAATGCTGGCCTTCATATCAACATGTCGC***CGGTTTATCCCTGCTGGCGCGGGGAACTC***GCCAAACGTGAGAAAAAATTCTCCAGACCTGA***CGGTTTATCCCTGCTGGCGCGGGGAACTC***GTTACATGGTCGCCCATTGCTGCCGGATTTAA***CGGTTTATCCCTGCTGGCGCGGGGAACTC***GCGACCGGGTTAACGCCATTGAGCGTGATAAG***CGGTTTATCCCTGCTGGCGCGGGGAACTC***CAGCTCCCATTTTCAAACCCATCAAGACGCGGTACCTTAATTAA*GAATTC*CGG

Note: Repeats are bolded and italicized, spacers are colored.

Brouns, S.J., Jore, M.M., Lundgren, M., Westra, E.R., Slijkhuis, R.J., Snijders, A.P., et al. (2008). Small CRISPR RNAs guide antiviral defense in prokaryotes. *Science* 321(5891)**,** 960-964. doi: 10.1126/science.1159689.
